# Beyond survival: reconstructing body image and femininity after breast cancer

**DOI:** 10.1590/1806-9282.723EDIT

**Published:** 2026-06-15

**Authors:** Amanda Piato, José Roberto Morales Piato, Bruna Salani Mota, José Roberto Filassi, Edmund Chada Baracat, José Maria Soares

**Affiliations:** 1 Universidade Santo Amaro, Faculty of Medicine – São Paulo (SP), Brazil.; 2 Universidade de São Paulo, Hospital das Clínicas, Faculty of Medicine, Department of Obstetrics and Gynecology, Mastology Sector, Gynecology Discipline – São Paulo (SP), Brazil.; 3 Universidade de São Paulo, Hospital das Clínicas, Faculty of Medicine, Department of Obstetrics and Gynecology – São Paulo (SP), Brazil.

Mastectomy is still indicated in specific situations, such as when the tumor is extensive or multifocal, or when the patient carries genetic mutations that increase cancer risk. Even in these cases, the contemporary perspective is different: we aim to preserve as much skin, areola, and nipple as possible, using implants or autologous flaps to reconstruct the breast in the same surgical procedure. Immediate reconstruction is preferable, as it prevents the psychological impact of sudden loss and reduces the need for future surgeries. Beyond restoring the body's contour, it helps a woman recognize herself again in the mirror, aiding her emotional and sexual recovery^1^.

Not all patients, however, require mastectomy. In a significant number of cases, it is possible to perform breast-conserving surgery, removing only the tumor and a small safety margin without compromising oncologic control. For this to be feasible, diagnosis must occur in the early stages of the disease, primarily through mammography. It is in this context that oncoplastic surgery represents a turning point. By uniting the principles of oncologic surgery with the techniques of plastic surgery, it allows for the removal of the tumor with safe margins while simultaneously reshaping the breast. More than simply preserving, oncoplasty often improves aesthetic outcomes by correcting asymmetries, elevating breast position, and providing a more harmonious shape than before the surgery^1^. This approach symbolizes a paradigm shift: breast cancer treatment does not have to leave marks of mutilation. It can, with skill and sensitivity, restore beauty and bodily equilibrium, strengthening self-esteem and the sense of femininity.

Reconstruction—whether following a mastectomy or an oncoplastic breast-conserving surgery—is not merely a question of appearance. It plays a central role in the recovery of self-esteem. The woman who goes through cancer experiences various losses: of her hair, her routine, her sense of predictability, and her own self-image. Recovering the breast is also about regaining a sense of wholeness, confidence, and the power to feel alive and desirable once more^2^.

Self-esteem does not mean denying the scars but reframing their meaning. They come to tell a story of overcoming, not of loss. The reconstructed woman is not a copy of who she was; she is a new version, conscious of her own strength^2,3^.

Female sexuality after breast cancer is a topic that still demands space and attentive listening. Physical, hormonal, and emotional changes can interfere with desire and intimacy. However, when there is support and open dialog, the body finds new pathways to pleasure^3^. Reconstruction and oncoplastic surgery play a crucial role in this process. By restoring harmony and confidence, they also reignite the connection with one's own body—a body that was wounded but is reborn with new forms and meaning^2,3^.

It is essential that oncological care includes conversations about sexuality, desire, and affection. To speak of pleasure is also to speak of life. The body image of a woman after cancer is made of scars, but also of rebirths^4^. Modern medicine understands that healing is not just about eliminating the disease, but about reconstructing meaning, identity, and a sense of belonging. Every patient who looks at herself with pride again is proof that, even after cancer, it is possible to feel whole, beautiful, and profoundly woman once more.

The path from breast cancer patient to empowered survivor is no longer defined by the disease alone, but by a modern commitment to healing that is as comprehensive as it is compassionate. Today's paradigm in surgical oncology, exemplified by oncoplastic techniques and immediate reconstruction, has elevated care beyond mere tumor eradication to actively restoring the body's harmony and a woman's self-image. This tangible restoration of form, however, is only the beginning. The true measure of success lies in the intangible yet vital reconstruction of confidence, femininity, and the capacity for pleasure—reclaiming the narrative from one of loss to one of profound resilience and renewal. It is this imperative to heal the whole person—body, identity, and spirit—that defines the highest standard of care, ensuring every woman can look toward a future not just of survival, but of wholeness and pride.

## Data Availability

The datasets generated and/or analyzed during the current study are available from the corresponding author upon reasonable request.
